# Evaluation of balance recovery stability from unpredictable perturbations through the compensatory arm and leg movements (CALM) scale

**DOI:** 10.1371/journal.pone.0221398

**Published:** 2019-08-28

**Authors:** Caroline Ribeiro de Souza, Marina Torres Betelli, Patrícia Sayuri Takazono, Julia Ávila de Oliveira, Daniel Boari Coelho, Jacques Duysens, Luis Augusto Teixeira

**Affiliations:** 1 Human Motor Systems Laboratory, School of Physical Education and Sport, University of São Paulo, São Paulo, Brazil Centre for Engineering, Modeling and Applied Social Sciences (CECS), Federal University of ABC, São Paulo, Brazil; 2 Centre for Engineering, Modeling and Applied Social Sciences (CECS), Federal University of ABC, São Paulo, Brazil; 3 Department of Movement Sciences, Faculty of Kinesiology and Rehabilitation Sciences, Catholic University of Leuven, Leuven, Belgium; Berner Fachhochschule, SWITZERLAND

## Abstract

Following unpredictable large-magnitude stance perturbations diverse patterns of arm and leg movements are performed to recover balance stability. Stability of these compensatory movements could be properly estimated through qualitative evaluation. In the present study, we present a scale for evaluation of compensatory arm and leg movements (CALM) in response to unpredictable displacements of the support base in the mediolateral direction. We tested the CALM scale for intra- and inter-rater reliability, correlation with kinematics of arm and leg movement amplitudes, and sensitivity to mode (rotation, translation and combined) and magnitude (velocity) of support base displacements, and also to perturbation-based balance training. Results showed significant intra- and inter-rater coefficients of agreement, ranging from moderate (0.46–0.53) for inter-rater reliability in the arm and global scores, to very high (0.87–0.99) for inter-rater leg scores and all intra-rater scores. Analysis showed significant correlation values between scale scores and the respective movement amplitudes both for arm and leg movements. Assessment of sensitivity revealed that the scale discriminated the responses between perturbation modes, platform velocities, in addition to higher balance recovery stability as a result of perturbation-based balance training. As a conclusion, the CALM scale was shown to provide adequate integrative evaluation of compensatory arm and leg movements for balance recovery stability after challenging stance perturbations, with potential application in fall risk prediction.

## Introduction

Unpredictable large-magnitude perturbations to stance correspond to some of the most critical circumstances threatening upright balance stability. This situation requires feedback-based identification of direction and magnitude of the perturbation to generate a scaled whole-body compensatory postural response in the short period available for balance recovery. An additional point challenging recovery of balance stability in this situation is that the primary (first trial) postural response has been shown to be compensated by generating exaggerated movements, leading to larger displacement of the center of mass (CoM) in comparison with the ensuing perturbations [1, 2]. Unsurprisingly, unpredictable balance perturbations in daily life, like erroneous body weight shift and tripping or stumbling, have been reported to be the most frequent cases leading to falls in at-risk older individuals [3]. Of particular concern regarding stance perturbations leading to falls is balance stability in the body frontal plane. Research has shown that amplitude of mediolateral (ML) spontaneous sway, amplified by lack of vision, is the single best predictor of future falling risk induced by mechanical perturbations in older individuals [4]. Additional investigation has shown that older individuals with history of falls have increased amplitude of ML sway either under full or occluded vision [5], and reduced functional limits of stability in the ML direction [6]. Although balance instability in the frontal plane is particularly critical to older persons, sudden unpredictable sideways displacements of the support base have been shown to lead to high incidence of falls even in healthy young individuals [7]. On the other hand, a recent systematic review and meta-analysis has indicated a lack of a single assessment tool able to predict with high certainty fall risk in older individuals [8]. From these findings, it is apparent the scarcity of a reliable evaluation tool of an individual’s capacity to recover balance stability following a threatening perturbation. Such a balance threatening context seems to require association between unpredictability and large magnitude of stance perturbations. Considering that postural responses to this kind of perturbation are featured by diverse arm and leg compensatory movements [9, 10], it would be desirable to count on an instrument evaluating integratively both upper and lower limb compensatory movements for balance recovery.

Previous investigation focusing on lower limb compensatory movements to stance perturbations has identified different movements apparently related to the degree of challenge to balance stability, ranging from fixed-support response to support-change strategies through single or multiple steps [9, 10]. A fixed-support response reflects the ability to recover balance from a perturbation by keeping CoM within the limits of balance stability over the unchanged support base area. To be observed, this reactive response requires instruction to “try not to step” in stance perturbations of large-magnitude [11, 12], and it is scaled to perturbation magnitude through differential activation of the agonist muscles [13]. The fact that in this reactive movement category body balance is recovered without any auxiliary leg displacement indicates high stability of balance recovery. In situations in which a perturbation leads to increased balance instability, so that an individual is unable to recover body balance after a perturbation by keeping the feet in place, one or multiple steps represent alternative compensatory strategies to prevent a fall [14]. Compensatory stepping reactions can be adjusted to perturbation magnitude by modulating metric parameters, like step length, or by using different stepping patterns [11]. For ML perturbations applied randomly to either side, single step responses have been characterized by a sidestep [15] or a large cross-over step with the swing foot being moved from one to the other body side, crossing either in front of or behind the support leg [11]. In instances in which a single step is insufficient to restore a safe stability margin of CoM over the support base at first foot contact, multiple steps are employed to recover balance stability [6]. The most frequent compensatory response pattern in these circumstances is featured by a sequence of sidesteps, with a small medial step followed by a longer lateral step with the opposite leg [15, 16]. From the aforementioned research, it appears that compensatory leg movements are generated according to the magnitude of stance perturbation by scaling a given movement pattern (e.g., step amplitude) or by employing different movement patterns associated with the degree to which balance stability was threatened (e.g., single X multiple steps). From this perspective, stability of balance recovery following an unpredictable perturbation might be inferred from the pattern and amplitude of compensatory leg movements.

Unpredictable stance perturbations have been shown to lead not only to leg but also to associated arm compensatory movements, with similar timing of muscular activation onset across the upper and lower limbs [17–20], suggesting a centrally multilimb coordinated response for balance recovery [21–23]. Compensatory arm movements to a perturbation have been evidenced to vary in amplitude depending on balance stability. First trial stance perturbations often evoke a primary arm reaction, which has been proposed to be the summation of a general startle reflex and a functional compensatory balance response [24]. Consistent with the notion that the primary arm movements are functionally modulated, research has shown that those reactions are scaled to perturbation magnitude [19], with arms’ amplitude [16] and velocity [25] being affected by perturbation direction. Arms’ movements can be used as a counterweight, being moved to the opposite direction of body disequilibrium in the search for stabilization of CoM over the support base [17]. Additionally, it has been shown that arms’ movements are used to exploit interjoint reaction torques, leading to increased moment of inertia of the body, and so increasing time available for balance recovery [12, 26]. A further compensatory arm movement pattern to large-magnitude stance perturbations consists of grasping reactions, which are frequently used when a support surface is available nearby [27]. Grasping reactions to balance perturbations are distinct from voluntary grasping in timing, with shorter initiation delay and movement time in comparison to the latter [28]. In the understanding of the functional role played by compensatory arm movements, it is relevant to consider that they seem to be used in coordination with compensatory leg movements, with interplay between the upper and lower limb reactions apparently having the common aim of recovering upright balance stability [22, 29]. Based on this conceptualization, it is plausible that stability of balance recovery from a perturbation is reflected in the spatial characteristics of compensatory arm movements.

Even though the literature on compensatory movements has described a variety of arm and leg movements to recover body balance following an unpredictable perturbation, less information is available on integration between compensatory arm and leg movements to respond to large-magnitude stance perturbations. As kinematic analysis requires expensive motion capture systems for such an evaluation, a tool is required to assess whole-body responses in different applied contexts. In the present study, we propose a scale for analysis of compensatory arm and leg movements (CALM), rating the upper and lower limb reactions to unpredictable perturbations as a function of associated balance recovery stability [9, 10]. The CALM scale was tested in the analysis of compensatory movements to perturbations in the ML direction on a moveable platform. To produce a context of unpredictability, we used randomized rotation, translation and combined rotation-translation of the support base to either side in variable platform velocities. By combining these factors, we conducted the scale assessment over a series of non-repeated (single) perturbations. In addition to intra- and inter-rater scale reliability, we tested correlation between CALM scale scores and limbs’ kinematics, assuming that lower scores in the scale should be associated with increased amplitudes of arm and leg movements, and vice-versa. Scale sensitivity to effects of reactive balance training [16, 30] was assessed by comparing results between trained and untrained individuals for the tested balance perturbations.

## Methods

### Participants

Evaluation of the CALM scale was based on data of 46 male and female healthy physically active participants, age range 18–35 years (*M* = 22.16, *SD* = 3.93)^1^. Half the participants were naïve for the probing stance perturbations (untrained group), and the other half experienced a sequence of stance perturbations before evaluation (trained group). Training was made through a sequence of 72 unpredictable stance perturbations of the support base in the ML direction. Half the participants in the trained group received the perturbation-based balance training in a blocked and half in a pseudorandom schedule in a single session. Training was provided with the purpose of inducing improvement of compensatory movements, and then testing the scale sensitivity to gains of reactive responses. Participants provided informed consent and experimental procedures were approved by the institutional review board in accordance with the declaration of Helsinki.

### Evaluation and instruments

Perturbations in both training and evaluation were applied automatically by means of a custom-made moveable platform controlled through a custom LabView computer interface (National Instruments (see [31] for details). The main purpose of our protocol was to evaluate compensatory arm and leg movements to large magnitude ML stance perturbations in the context of unpredictable mode, direction, magnitude and time of perturbation. The platform moved through a single axis. Participants were positioned on the platform so that potential disequilibrium would take place in the frontal plane of the body. Mediolateral perturbations were applied in three modes: rotation, translation or combined rotation-translation, to either side, in three platform peak velocities: 20°/s / 20 cm/s (low), 30°/s / 30 cm/s (intermediate) or 40°/s / 40 cm/s (high), keeping peak acceleration of 500^o^/s^2^ / 500 cm/s^2^ and displacement amplitude of 7^o^ / 7 cm constant across perturbations. The factors perturbation mode (3), direction (2) and velocity (3) of platform displacement were combined to generate 18 distinct stance perturbations. To create the context of unpredictability in the evaluation trials, perturbations were pseudorandomly sequenced (same sequence across participants). No cueing was provided about perturbation onset time, with trials triggered randomly between 2–5 s following a verbal prompt. With these procedures, we prevented adaptation between trials due to repeated exposure to the same perturbation, pre-planning of the ensuing movement, or anticipation of platform movement onset. Thus, our protocol required pure reactive responses based on different sources of on-line feedback on the effect of the perturbation on body balance. The initial participant’s posture on the platform was keeping the Romberg’s stance, with the feet oriented forward touching each other, maintaining both arms relaxed hanging beside the trunk, and palms of the hands lightly touching the upper legs. The reduced ML support base was employed to impose a high challenge to balance recovery. Balance perturbations were applied while the participant gazed at a frontal spot positioned at the eyes height.

To become aware of the stance perturbations, participants watched a video demonstrating a person responding to the different platform perturbations included in the protocol. In the videos, the participant observed feet-in-place responses for all modes of perturbation. This procedure is thought to have been particularly relevant for the untrained group, so that they were aware of all kinds of perturbation used for evaluation without experiencing them. Absence of familiarization trials allowed for evaluation of the primary response to each single perturbation in the untrained group, in comparison to the trained group previously exposed to the perturbations. After video watching, subjects were warmed up for 5 min. with global movements. Initial feet position was marked on the support base with adhesive tapes (5-cm width). Other adhesive tape marks were fixed 15 cm away from the outer border of the feet to either side, in parallel to the feet orientation. The tapes were used for reference of leg movement amplitude for evaluation through the scale.

To prevent falls, participants wore a safety harness supported by two ropes tied at the shoulders height with the other end attached overhead. The safety harness was adjusted so that it would support participants’ body in the case of falls, but not providing any support while they stood on the platform before perturbation or during balance recovery without falls. The participant’s aim across perturbations was to recover balance after support base displacements trying to maintain the initial body posture. They were instructed that if unable to recover balance stability after a perturbation by keeping the initial posture, arm and leg movements could be used to recover a stable upright stance. Additionally, they were instructed to refrain from grasping the safety ropes unless they were unable to recover balance using other resources, with this response being considered as near fall.

Participants’ responses were filmed from behind using a commercial digital camera (Sony), sample rate of 60 Hz, for off-line analysis based on the CALM scale. For kinematic analysis of amplitude of upper and lower limb movements, reflective markers (14 mm diameter) were attached bilaterally on the body at the following points: (a) acromion, (b) humeral trochlea, (c) midway between anterior superior iliac spine and midline, and (d) calcaneus. Kinematic markers were tracked through four optoelectronic cameras (Vicon, Nexus T10), at the sample rate of 200 Hz.

### Development of the compensatory arm and leg movements (CALM) scale

The CALM scale was developed with the purpose of being an integrative instrument to evaluate the different compensatory arm and leg movements in response to unpredictable large magnitude stance perturbations. It was elaborated across three stages: (1) Compilation of arm and leg compensatory movements described in the literature, and those identified in the participants’ responses to mediolateral stance perturbations we applied for evaluation (described in the following). (2) Assignment of scores to the distinct compensatory movement patterns. Scores were ascribed to arm movements based on amplitude of hands’ displacement, with lower scores for wider movements away from the initial position. For leg movements, we ascribed scores based on the pattern and amplitude of motions. Based on literature [9, 10] and our own preliminary analysis of the current data, we defined the following sequence of scores for balance recovery stability: feet-in-place > feet sliding > leg swing > single step > multiple steps. The patterns of leg swing, single and multiple steps were differentiated as a function of movement amplitude (see description in the ensuing paragraph). (3) Raters training to use the scale. In this latter stage, the raters scored individually all probing responses of 20% of participants (10 of each group), and compared the results. Cases of disagreement between the raters were discussed for reaching a consensus.

For both arm and leg compensatory movements, the lowest score (1) was given for the most unstable response of being supported by the safety ropes (grasping or hanging), independent of leg movements, which were classified as near falls. Arm movements were classified as a function of their amplitudes (the greater right/left amplitude of hand motion was used for scoring), as described in the following (score in parenthesis): *Large* (2), large shoulder abduction raising the hand(s) at or above the shoulders height. *Moderate* (3), moderate shoulder abduction raising the hand(s) in the range between about 10-cm lateral distance from the initial position and below the shoulders’ height. *Small* (4), minor shoulder abduction, with contact loss between the hand(s) and the leg(s) less than about 10 cm. *Motionless* (5), the most stable response, maintaining the hands in contact with the legs while recovering body equilibrium.

**Fig 1:**
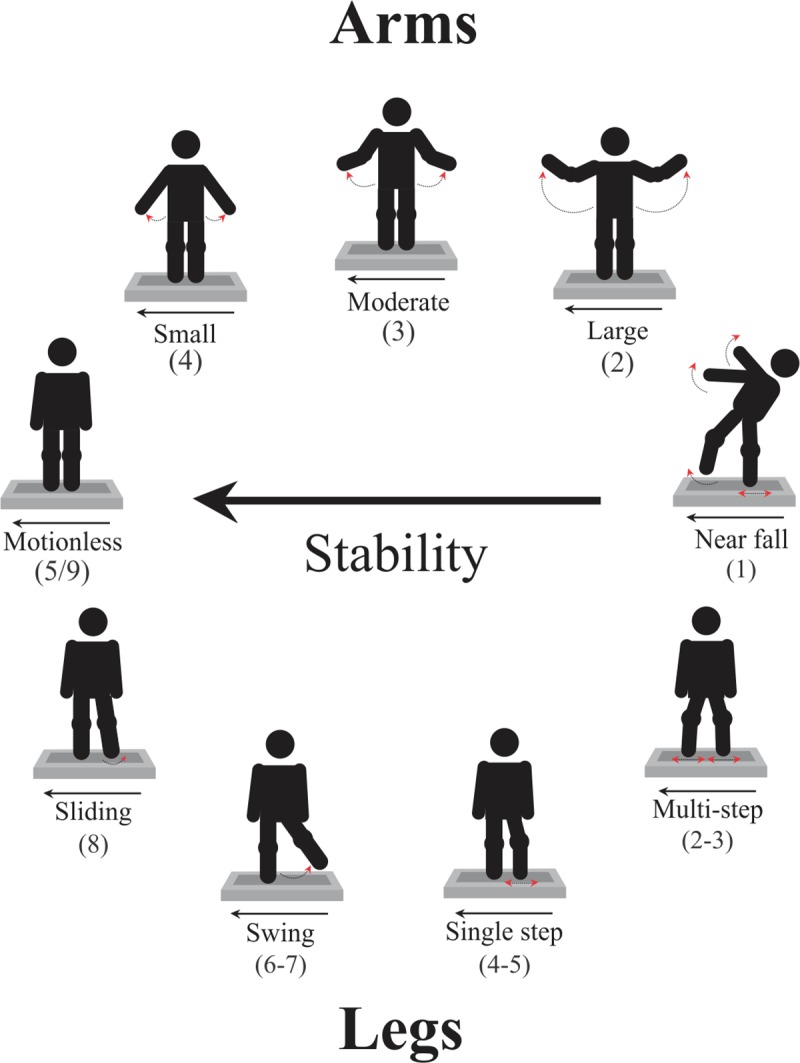
Arm-leg compensatory movements. Representation of arm (upper sequence) and leg (lower sequence) compensatory movement patterns observed in response to unpredictable base of support displacement. The compensatory movements are organized by response stability (from right to left), ranging from the most stable “motionless” to the less stable “near fall” responses (grasping the safety ropes), with scores presented in parenthesis below the respective movement pattern. Variation of leg movement amplitudes used for rating through the scale is not represented.

Compensatory leg movements were classified as follows (score in parenthesis): *Multiple steps* (2–3), changing the support base for balance recovery through two or more steps; this classification was used independent of the multi-stepping pattern presented (see Introduction). *Single step* (4–5), balance recovery through a single step; this classification was used independent of whether the participant used a lateral sidestep (by moving the leg loaded by the platform motion) or stepped by crossing over (in front of or behind) the support leg. *Leg swing* (6–7), featured by outward swinging one leg for counter-weighting lateral body leaning while supporting the whole body on the other leg. These three categories were subdivided into “large” (lower score) or “small” (higher score) amplitudes, respectively for leg movements crossing the 15-cm mark on the platform and for leg movements beyond the 5-cm mark but not crossing the 15-cm mark on the platform. *Sliding* (8), featured by short one-foot or two-feet outward sliding over the support base (no feet-ground contact loss), or short (few centimeters) one-foot rising above the ground landing at about the place. *Motionless* (9), balance recovery keeping the feet in place. A representation of the distinct arm and leg compensatory movement patterns is shown in Fig 1, organized as a function of their stability for balance recovery, with scores presented in parenthesis below the respective movement pattern (see guidelines for application of the CALM scale at dx.doi.org/10.17504/protocols.io.2p2gdqe).

### Data analysis

Scale reliability, correlation with kinematic data and sensitivity were assessed based on scores achieved in each one of the 18 probing trials for participants of both the trained and untrained groups. Intra- and inter-rater scale reliability was assessed from analyses of two raters (PST and CRS), who were blind for group composition, performing the evaluation of the whole set of trials for both arm and leg movements of a sample of 12 participants (6 from each group), selected by means of computer-generated random numbers. As each participant was probed in 18 trials, we evaluated in total 216 responses. Intra- and inter-rater reliability analysis was made through Cohen’s kappa coefficients. For intra-rater reliability analysis, we used a two-week interval between videotape assessments.

For correlational assessment, separate scores of arm and leg movements were correlated to kinematic measurements of respective movement amplitudes. For arm movements, the scale scores were correlated with maximum amplitude of shoulder abduction angle, given by the vectors formed by the shoulder-elbow and shoulder-hip markers. For leg movements, the scale scores were correlated with maximum amplitude of hip abduction angle, calculated by the angle formed between the absolute vertical axis and the vector formed by hip-calcaneus of the swing leg. Kinematic data were digitally low-pass filtered with a cut-off frequency of 10 Hz through a dual-pass fourth-order Butterworth filter. Data processing was made through a Matlab (MathWorks) routine after visual data inspection. Spearman’s rho correlation coefficients (*r*_s_) were used to analyze association between scale scores (averaged between raters) and kinematic data.

For assessment of scale sensitivity to perturbation mode, velocity, and balance stability gain from perturbation-based balance training, we averaged scores between the right and left sides for homologous perturbations (same mode and velocity), given that scores were equivalent between sides (see Results). Analysis was performed through pairwise comparisons, using the Mann-Whitney U test for group-related comparisons in each perturbation mode by velocity, and the Wilcoxon matched pairs test for effects of perturbation mode (rotation X translation X combined; three velocities averaged), and velocity (low X high^2^; separately for each perturbation mode). Statistically significant effects are reported only.

Statistica software (StatSoft, Tulsa, UK) was used for all analyses. Images representative of the different compensatory movements, raw data and results from analyzes and images of different patterns of compensatory arm and leg movements are available for open access at http://dx.doi.org/10.17632/fjm652j7gf.1#folder-bcd59584-079d-4951-8a0f-106e80f2a9a8.

## Results

As we expected no effects of perturbation side, a preliminary analysis was performed to compare perturbations to the right versus the left side. The average scores of perturbations for each side were compared through the Wilcoxon matched pairs test. Results indicated no statistical significant difference, *Z* = 0.23, *p* > 0.8. In the following analyses we present data collapsed between the two perturbation sides.

**Fig 2:**
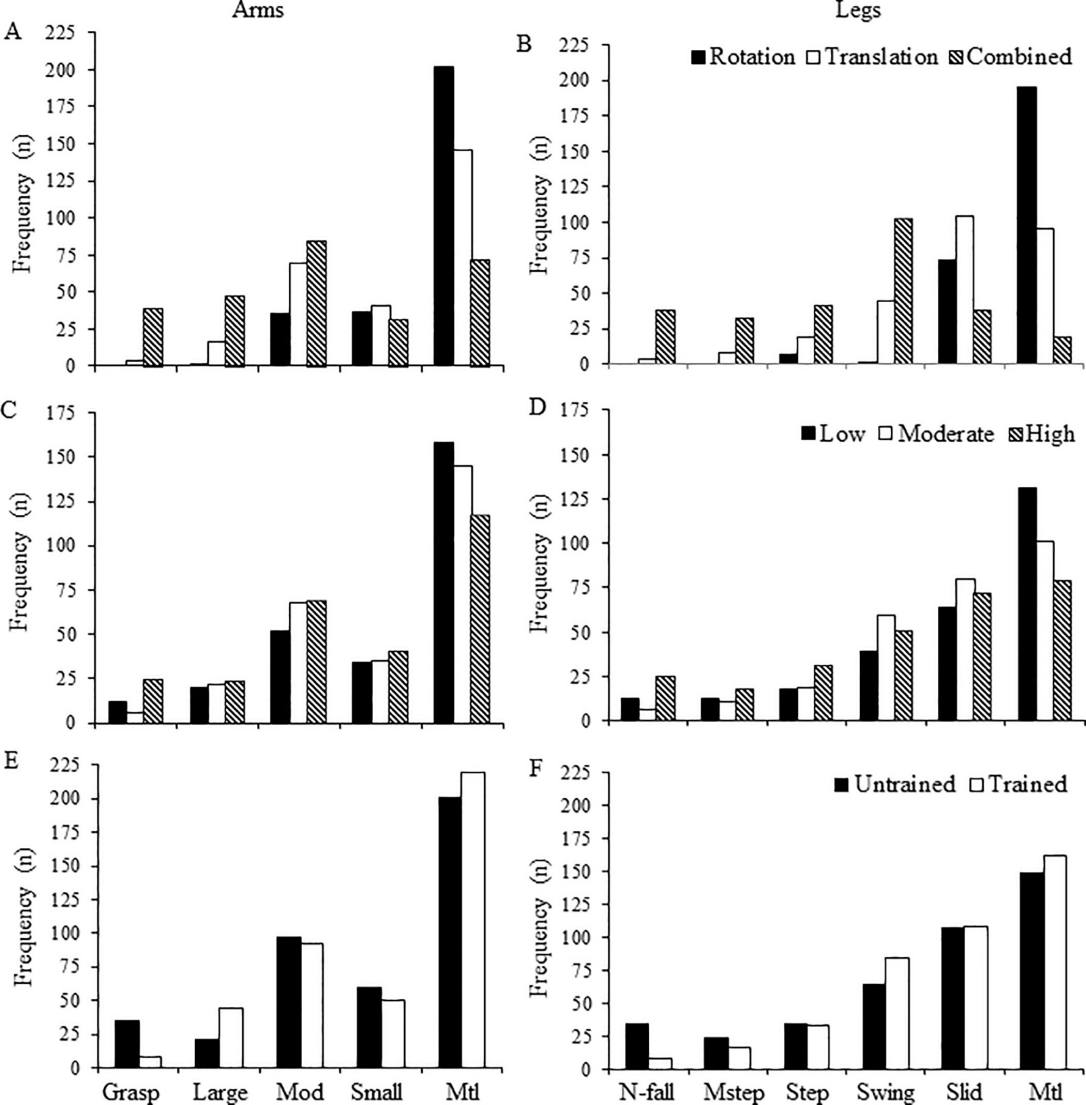
Frequency of compensatory movement categories. Absolute frequencies of categories of arm (left-sided panels) and leg (right-sided panels) compensatory movements. Data were collapsed between perturbation sides, and the frequencies are shown separately as a function of perturbation mode (A-B), velocity (C-D), and training (E-F). Abbreviations: Mod = moderate, Mtl = motionless, N-fall = near fall, Mstep = multiple steps.

In Fig 2 we present absolute frequencies of the arm (left-sided panels) and leg (right-sided panels) compensatory movement categories, separately for perturbation mode (A-B), velocity (C-D), and training (E-F). As that figure shows, our perturbation protocol elicited diversified compensatory arm and leg movements. It is worth noticing that we observed two patterns of compensatory leg movements not previously reported in the literature, namely leg swing and feet sliding. In the former, the swinging leg was used as a counterweight to compensate for trunk sway to the opposite side, while the latter led to a small enlargement of the support base. This descriptive analysis shows whole body responses, suggesting that arm and leg compensatory movements were associated, being affected equivalently by perturbation type, velocity and training.

### Reliability

For analysis of intra-rater reliability, the proportions of between-evaluation (day 1 X day 2) agreement were as follows: Rater 1: arms = 98.61%, legs = 98.61%, and global score = 97.22%; Rater 2: arms = 98.61%, legs = 99.54%, and global scores = 98.15%. Cohen’s kappa analysis indicated coefficients ≥ 0.97 for both raters (Table 1). For analysis of inter-rater reliability, proportions of agreement were as follows: Day 1: arms = 65.74%, legs = 90.28%, and global score = 60.19%; Day 2: arms = 62.96%, legs = 90.74%, and global score = 57.41%. Cohen’s kappa coefficients range was 0.46–0.88 (Table 2). Based on the Cohen’s [32] proposed interpretation, these agreement coefficients ranged from moderate (0.41–0.60) for inter-rater reliability in the arm and for global scores to very high (0.81–1.00) for inter-rater leg score analysis and all intra-rater scores. All partial and overall coefficients were statistically significant (*p* < 0.01).

**Table 1:** Cohen’s kappa coefficients for intra-rater reliability.

	**Rater 1 (Day 1 x Day 2)**				**Rater 2 (Day 1 x Day 2)**			
	Range	Overall	*Z*	*SE*	Range	Overall	*Z*	*SE*
Arms	0.97–1.00	0.98	22.84	0.04	0.96–1.00	0.98	20.11	0.04
Legs	0.97–1.00	0.98	28.17	0.03	0.97–1.00	0.99	29.03	0.03
Global	0.91–1.00	0.97	35.42	0.03	0.94–1.00	0.98	33.93	0.03

Note: Range of partial coefficients across compensatory movement patterns, overall coefficient across scores, *Z* value, and standard error (*SE*).

**Table 2:** Cohen’s kappa coefficients for inter-rater reliability.

	**Day 1 (Rater 1 x Rater 2)**				**Day 2 (Rater 1 x Rater 2)**			
	Range	Overall	*Z*	SE	Range	Overall	*Z*	SE
Arms	0.34–0.90	0.49	22.84	0.04	0.29–0.90	0.46	20.11	0.04
Legs	0.68–1.00	0.87	28.17	0.03	0.65–1.00	0.88	29.03	0.03
Global	0.31–1.00	0.53	35.42	0.03	0.27–1.00	0.50	33.93	0.03

Note: Range of partial coefficients across compensatory movement patterns, overall coefficient across scores, *Z* value, and standard error (*SE*).

### Correlational analysis with movement kinematics

**Fig 3:**
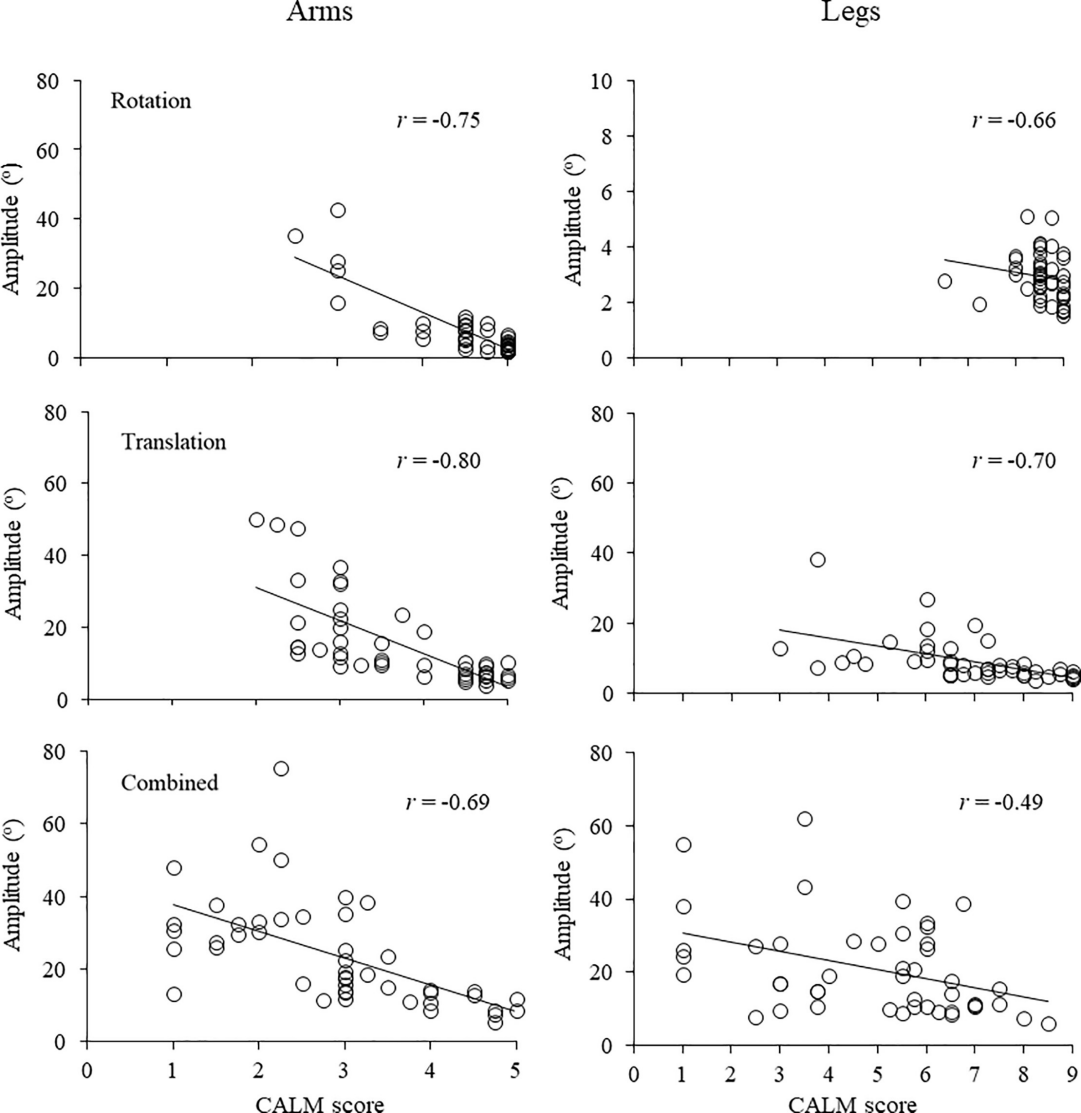
Correlation of CALM scale score and movement amplitude. Scatter plots and *r*_*s*_ values for correlations between CALM scale scores and respective movement amplitude based on kinematics for arm (left column) and leg (right column) movements, representing high velocity perturbations in the rotation (upper panels), translation (intermediate panels) and combined (lower panels) modes of perturbation. Data were collapsed between the groups and perturbation sides.

In Table 3 we present Spearman’s rho coefficients (*r*_s_) between scale scores and respective movement amplitudes (from kinematics) for arm and leg movements, separately for perturbation mode and velocity (data were collapsed between the two groups and the two sides). Results revealed correlation coefficients in the range of -0.48 to -0.81, with significant values in all analyses (*p* < 0.01). In Fig 3 we depict a graphic representation of a sample of those data, showing scatter plots for arm and leg movements in the high velocity for the three modes of perturbation. In this figure, one can see the association between angular amplitude of arm/leg movements and the corresponding scale-based scores. These results support the conclusion that the qualitative CALM scale scores are in agreement with objective measurements given by a gold standard reference as provided by kinematic analysis.

**Table 3:** Spearman’s rho coefficients (*r*_s_) between the CALM scale scores and respective movement amplitudes.

	Rotation			Translation			Combined		
	Low	Int	High	Low	Int	High	Low	Int	High
Arms	-0.81	-0.67	-0.75	-0.63	-0.81	-0.80	-0.72	-0.79	-0.69
Legs	-0.54	-0.57	-0.66	-0.53	-0.60	-0.70	-0.48	-0.49	-0.49

Note: Data for arms and legs are presented separately for perturbation mode by velocity (low, intermediate (Int) and high) collapsing data between the two groups and the two sides; all correlation values were statistically significant (*p* < 0.01).

### Sensitivity

**Fig 4:**
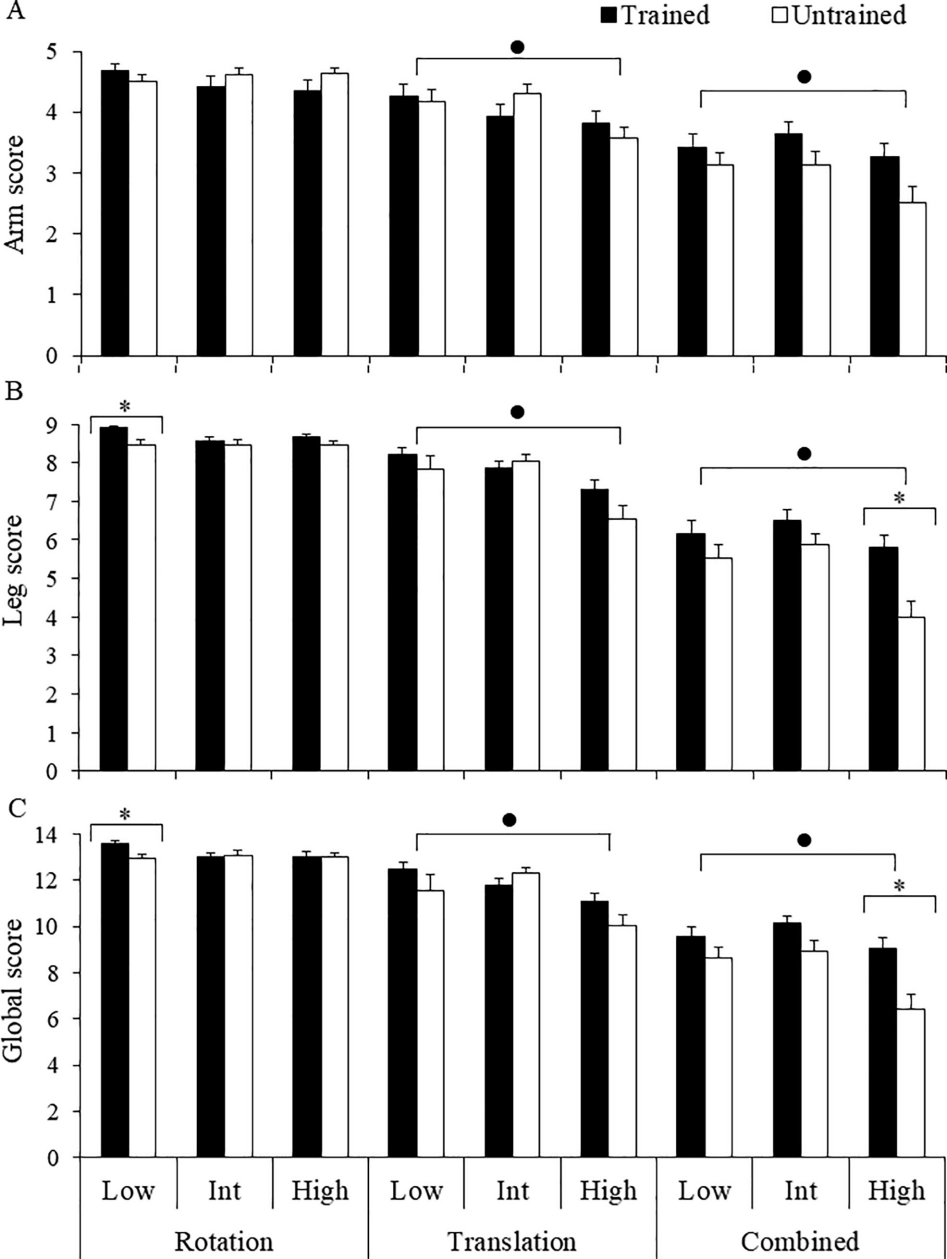
Comparison between the trained and untrained groups. Mean (SE in vertical bars) CALM scale scores for arm (A) and leg (B) compensatory movements, and for the global score (C). Data are presented for rotation, translation and combined perturbations by velocity: low, intermediate (Int) and high, averaged between perturbation directions. Asterisks represent significant differences between trained and untrained groups in specific mode by velocity perturbations, and filled dots represent significant differences between low and high velocity.

Analysis of scale sensitivity was made separately for arm and leg movements, and also for the global score. Results for arm movement scores (Fig 4A) revealed significant differences between the three perturbation modes, *Z* values range = 4.96–5.78, *p* values < 0.01, with scores organized in the following order: rotation > translation > combined. Comparisons between platform displacement velocities showed significantly higher scores for the low as compared to the high velocity in translation, *Z* = 4.19, *p* < 0.01, and combined, *Z* = 2.82, *p* < 0.01, perturbation modes. The effect of balance training was observed in the most challenging combined high velocity perturbation, *Z* = 2.17, *p* = 0.03, with higher scores for the trained than for the untrained group.

Results for leg movement scores (Fig 4B) revealed similar effects as found for arm movement scores. Analysis indicated significant differences between the three perturbation modes, *Z* values range = 5.11–5.91, *p* values < 0.01, with scores in the following relationship: rotation > translation > combined. Analysis of platform displacement velocities showed significantly higher scores for the low as compared to the high velocity in translation, *Z* = 4.22, *p* < 0.01, and combined, *Z* = 2.87, *p* < 0.01, perturbation modes. Analysis of effect of balance training indicated higher scores for the trained as compared to the untrained group in combined high velocity perturbations, *Z* = 3.02, *p* < 0.01.

Analysis of the global score (Fig 4C) showed similar sensitivity as that observed for the segmental scores. Results corroborated the same relationship of scores across perturbation modes: rotation > translation > combined, *Z* values range = 5.44–5.91, *p* values < 0.01. Significantly higher scores for the low as compared to the high velocity were found in translation, *Z* = 4.17, *p* < 0.01, and combined, *Z* = 3.08, *p* < 0.01, perturbation modes. Effect of balance training was observed in higher scores for the trained as compared to the untrained group in combined high velocity perturbations, *Z* = 2.92, *p* < 0.01. Overall, analyses revealed consistent effects of perturbation mode, perturbation velocity, and perturbation-based balance training across arm, leg and global scores, showing that the CALM scale was sensitive to the three factors manipulated.

## Discussion

In the present study, we developed and tested for reliability, correlation with limbs’ kinematics and sensitivity of the CALM scale for assessment of arm and leg compensatory movements to large-magnitude stance perturbations in the ML direction. A particular point worth mentioning in the protocol used was the context of unpredictable mode, direction, velocity and time of a series of support base displacements. With this protocol, we applied a sequence of unique perturbations requiring pure reactive compensatory movements, preventing between-trial adaptation and anticipatory postural adjustments [33]. Observation of about 40 cases of near falls in our results with young participants illustrates the challenge imposed to balance recovery in our perturbation protocol. Additional challenges to recover balance after perturbations in the current investigation were the biomechanical constraint of standing on a narrow support base, and the task constraints of avoiding stepping and grasping reactions. It should be noticed that, from the imposed constraints, the compensatory movements for balance recovery were not completely spontaneous, since the primary movements selected in similar situations are most frequently the easier and safer stepping or grasping reactions [10]. On the other hand, the instructions to strive to keep the initial posture, and grasping the safety ropes only as the last response resource, allowed for a clearer interpretation of stability of the observed compensatory movements regarding unconstrained evaluations in which those responses could be pre-planned. The context of perturbation unpredictability can be thought as corresponding to some of the most challenging daily living situations requiring fast reactive balance recovery of balance stability.

### Arm and leg compensatory movements

As expected, results showed that the sequence of perturbations elicited a variety of compensatory arm and leg movements. For arm movements, we observed higher frequencies of “motionless” responses for rotations and translations, while for the most challenging combined perturbations compensatory movements of moderate amplitude prevailed. Frequency of motionless arm responses seems to have been associated with the degree of challenge to balance recovery, with progressive lower values across the rotation, translation and combined perturbations. On the other hand, arm movements of moderate and large displacement amplitudes (see movements’ description in “Evaluation and instruments”), as well as grasping reactions, were found to be more frequent in combined perturbations. These results support the interpretation that compensatory upper limb movements were selected as a function of the magnitude of challenge to balance recovery imposed by the perturbation mode. One point to be observed in this regard is that the safety ropes were graspable above the shoulders. This feature may have induced part of the moderate and large amplitude movements of the arms, possibly aiming to grasp the ropes at movement onset.

Analysis of leg movements led to description of two categories not identified previously in the literature, namely “feet sliding” and “leg swing”. Fast outward feet sliding movements lead to enlargement of the support base regarding the restrictive initial Romberg posture. One-legged outward swing, while supporting body weight on the contralateral leg, can prevent loss of balance by using the swinging leg as a counterweight to trunk leaning toward the opposite direction. It is worth noticing that leg swing was prevalent across categories of compensatory movements for the most challenging combined perturbations. Thus, leg swing can be thought to be a frequent pattern of leg movements in response to high challenge balance perturbations when stepping is constrained. Both feet sliding and leg swing movements may have been specific to some extent to the constraints of an initial narrow support base associated with the instruction to try not to step in response to perturbations. Multiple steps were observed in several trials for combined perturbations, showing that this is a compensatory movement used not only by older [15] but also by young individuals when the perturbation is challenging enough. Distribution of the diverse patterns of compensatory leg movements across the perturbation modes suggests that they are adaptive to the specific challenge imposed to balance recovery. For the less challenging support base rotations, participants were able to attend the task constraint and recover balance through fixed-support responses in most trials, with rare cases of leg swing or stepping, and no occurrence of near falls. Conversely, compensatory leg movements ranging from leg swing to multiple steps, as well as near falls, were found to be more frequent in combined perturbations. Support base translations were found to lead to a profile intermediate between rotation and combined perturbations, with most responses featured by no or minimal (sliding) feet motion, but with several cases of less stable compensatory movements. Overall, this descriptive analysis supports the interpretation that the diverse compensatory arm and leg movements are flexibly selected to generate whole-body responses [21] as a function of the challenge imposed by a large-magnitude unpredictable perturbation to balance recovery.

### Scale evaluation

Analysis of CALM scale scores revealed that they were consistent with the challenge imposed by the different perturbation categories, with higher scores for rotations, intermediate for translations and the lowest scores for combined perturbations for both arm and leg movements. Scale sensitivity to the effect of perturbation magnitude was evidenced from lower scores in the high as compared to the low platform velocity in translation and combined perturbations. Lack of velocity-related effects in rotations, allied to very high scores in this perturbation mode, suggests that this kind of perturbation leads to subtle and less variable reactive responses across platform velocities. Accordingly, this perturbation mode seems to be less suitable for evaluation through the CALM scale. Scale sensitivity was also demonstrated for the effect of perturbation-based balance training. Achievement of higher scores by the trained in comparison with the untrained participants in the most challenging combined perturbations is consistent with the expected improved performance due to training of reactive postural responses [16, 30]. Observing higher scores for the trained group in the most challenging perturbations suggests that improved balance stability acquired through perturbation-based balance training is associated with compensatory upper and lower limb movements. From this comment, it seems the CALM scale is able to differentiate balance recovery stability in high but in not in low challenge stance perturbations. Consistent effects between the arm and leg scores suggests that either component of the CALM scale could be used separately with equivalent efficacy in the evaluation of balance recovery stability.

Evaluation of the CALM scale revealed significant correlations between both arm and leg movement scores with kinematic measurements of respective movement amplitudes in all (mode by velocity) forms of perturbation. Correlation coefficients between -0.48 to -0.81 can be considered to be moderate to high across perturbations (mode by velocity). By considering that compensatory arm and leg movements receiving lower scores can be expected to be performed usually through wider lateral motions, and vice-versa for higher scores, evaluation referenced to limbs’ kinematics is thought to be an objective indicator of a component differentiating some inter-movement categories (e.g., feet-in-place X sliding X swing/single step) and intra-movement categories (e.g., large X small for sliding, swing and single step movements). However, for the category of multiple steps one could not assume that the CALM score is associated with movement amplitude, given that multiple steps are usually individually shorter in amplitude than large swing movements and single steps. This corresponds to a limitation in analysis for leg movements based on angular kinematics of movement amplitudes. This limitation might underlie the finding of some moderate rather than high correlation values.

Analyses of intra- and inter-rater reliability showed moderate to very high coefficients of agreement. These reliability coefficients are a further positive point in the CALM scale testing, showing that the evaluation criteria have the required objectivity to lead to reproducible scores over time for a single rater, and to a high frequency of scores’ coincidence between raters. However, the reported values should be considered in the context that the raters participated in the scale development, extensively discussing the criteria for classification of the compensatory movements. Hence, extensive training may be required to achieve similar rates of coincident scores.

As a further comment about the generalizability of the reported resuts, the fact that our protocol provoked many cases of near falls in healthy young participants may lead to a floor effect in the evaluation of people with increased fall risk. It is possible that the platform velocity may have to be reduced in the assessment of fragile older or neurologic individuals to set appropriately the range of perturbation magnitudes for this group. Further research is needed to explore this point.

## Conclusions

Capacity to recover balance stability has been proposed to be properly evaluated in contexts of unpredictable large-magnitude stance perturbations [10, 17]. In the current study, we presented and evaluated the CALM scale for analysis of different compensatory arm and leg movements generated in response to perturbations with diverse degrees of challenge for balance recovery. Different from other scales rating performance in a set of quiet or dynamic balance tasks, intended to assess balance impairments in older or neurologic damaged individuals [34–36], our scale rates the diverse set of compensatory movements based on stability of balance recovery. Results showing (a) correlation of the scale scores with arm and leg movement kinematics, (b) sensitivity to the mode and magnitude (velocity) of perturbation and also to the effect of perturbation-based balance training, in addition to (c) high intra- and inter-rater reliability, indicate the suitability of the proposed scale for evaluation of compensatory limb movements in response to unpredictable large-magnitude stance perturbations as an index of balance recovery stability. We originally described two categories of compensatory leg movements, leg swing and feet sliding, which compose along with stepping movements the repertoire of lower limb compensatory movements for balance recovery. An additional innovative point in the CALM scale is the integrative evaluation of arm and leg compensatory movements, allowing for analysis of whole-body responses to stance perturbations. Moreover, to the best of our knowledge, this is the first scale for analysis of reactive postural responses for evaluation of balance recovery in healthy young individuals. As the CALM scale’s scores are proposed to be representative of the individual’s balance resilience to unpredictable large-magnitude perturbations, it can reveal to be an instrument potentially able to predict with high certainty the probability of falls in healthy young individuals.

### Notes

1. Data analyzed in this study were extracted from two unpublished investigations assessing the effect of training and previous experience on large-magnitude balance perturbations.
2. For the sake of simplicity, the intermediate velocity was excluded from analysis.

## Supporting information

S1 File CALM scale application guidelines with graphic representation, description of arm and leg movements and the respective scores. (DOCX)
