# Supplementary material for: Evaluation of balance recovery stability from unpredictable perturbations through the compensatory arm and leg movements (CALM) scale
Source: PLoS One. 2019 Aug 28;14(8):e0221398. doi: 10.1371/journal.pone.0221398 (PMC6713348; doi:10.1371/journal.pone.0221398)
Supplement: S1 File — (DOCX) [file pone.0221398.s001.docx]

| Compensatory Arm and Leg Movements (CALM) Scale | | |
| --- | --- | --- |
| Application guidelines  The compensatory arm and leg movements (CALM) scale is applied for analysis of stability of patterns of reactive responses to unpredictable large-magnitude stance perturbations in the mediolateral direction. Its application should be associated with the instruction to try not to step in response to perturbations, and keeping the feet together (Romberg posture) while waiting for the upcoming perturbation. Arm and leg movements are rated separately. Consider the wider movement between the right and left arms for rating. | | |
| Arm movements | | |
| Representation | Score | Description |
| 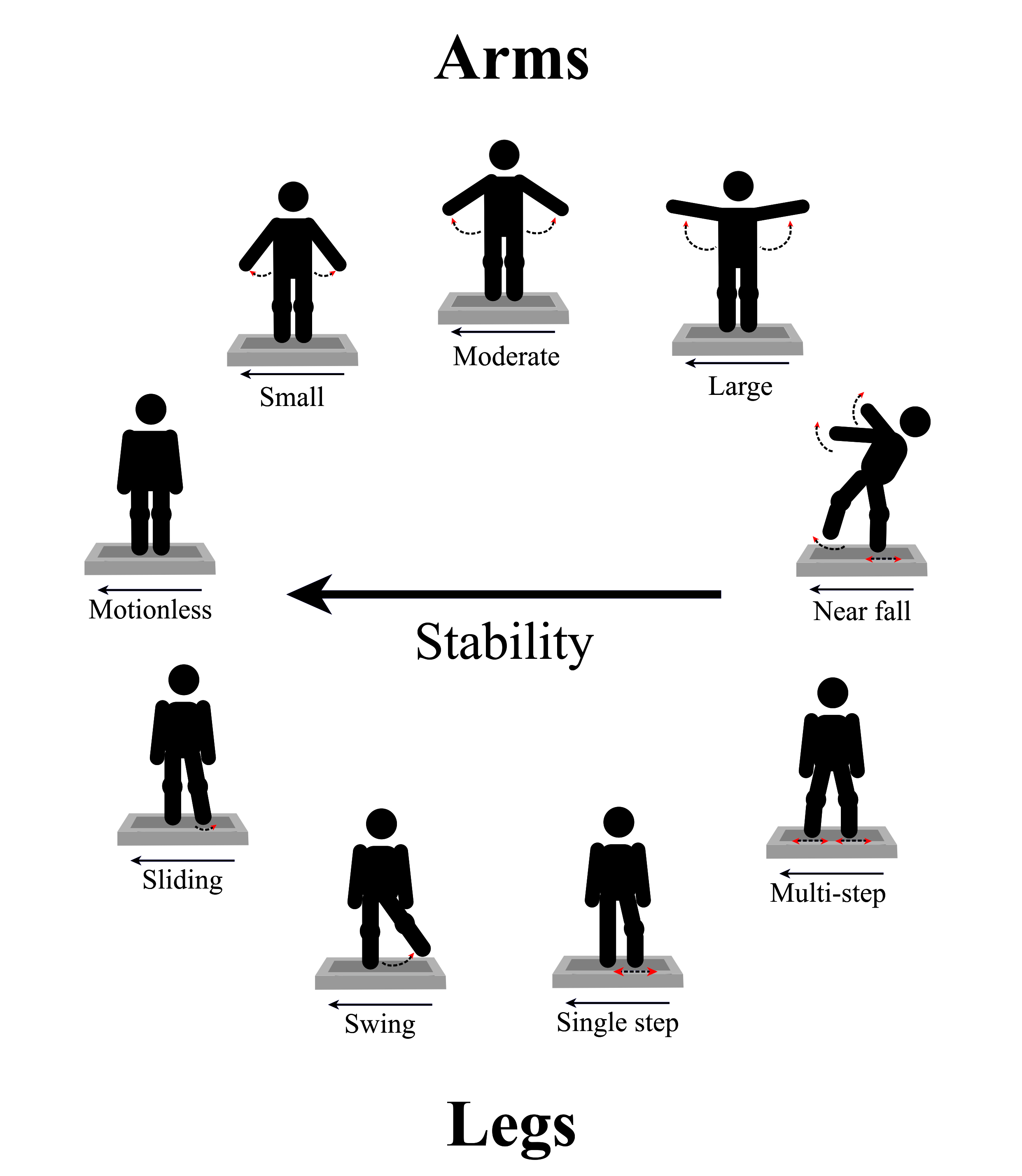 | 1 | **Near fall**. Being supported by the safety device or grasping a near surface. |
| 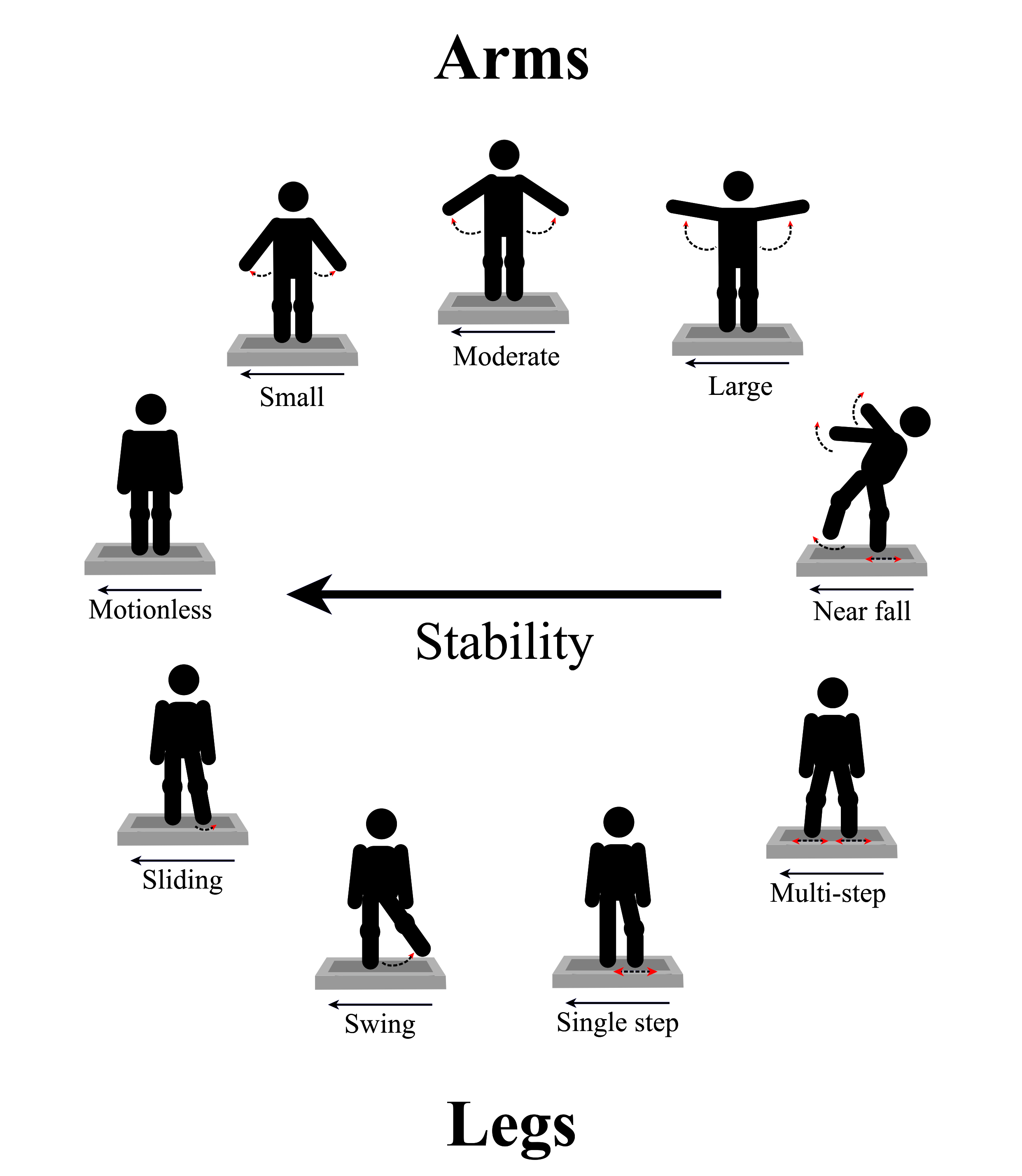 | 2 | **Large amplitude**. Large shoulder abduction raising the hand(s) above the shoulders height. |
| 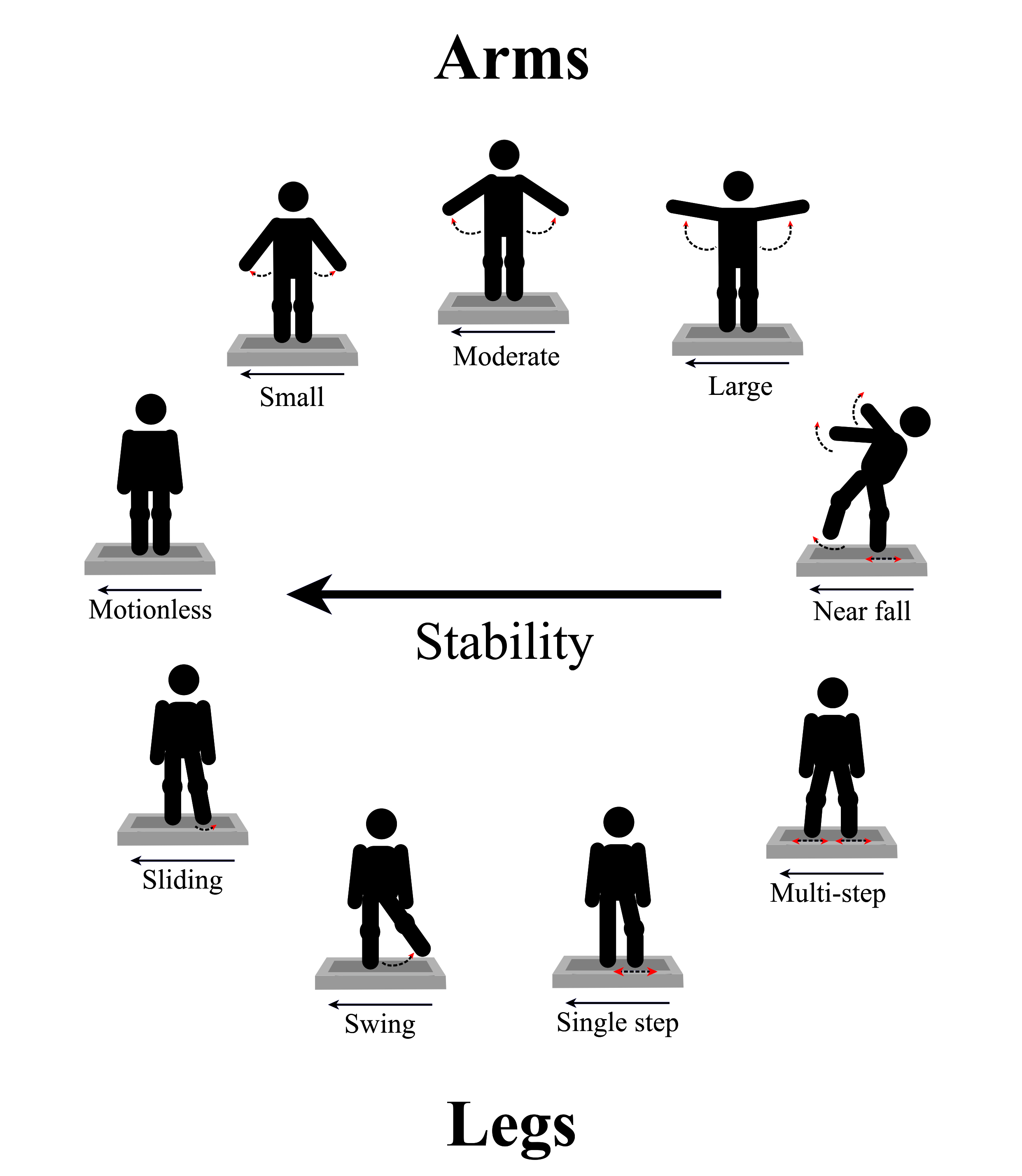 | 3 | **Moderate amplitude**. Moderate shoulder abduction raising the hand(s) below the shoulders height. |
| 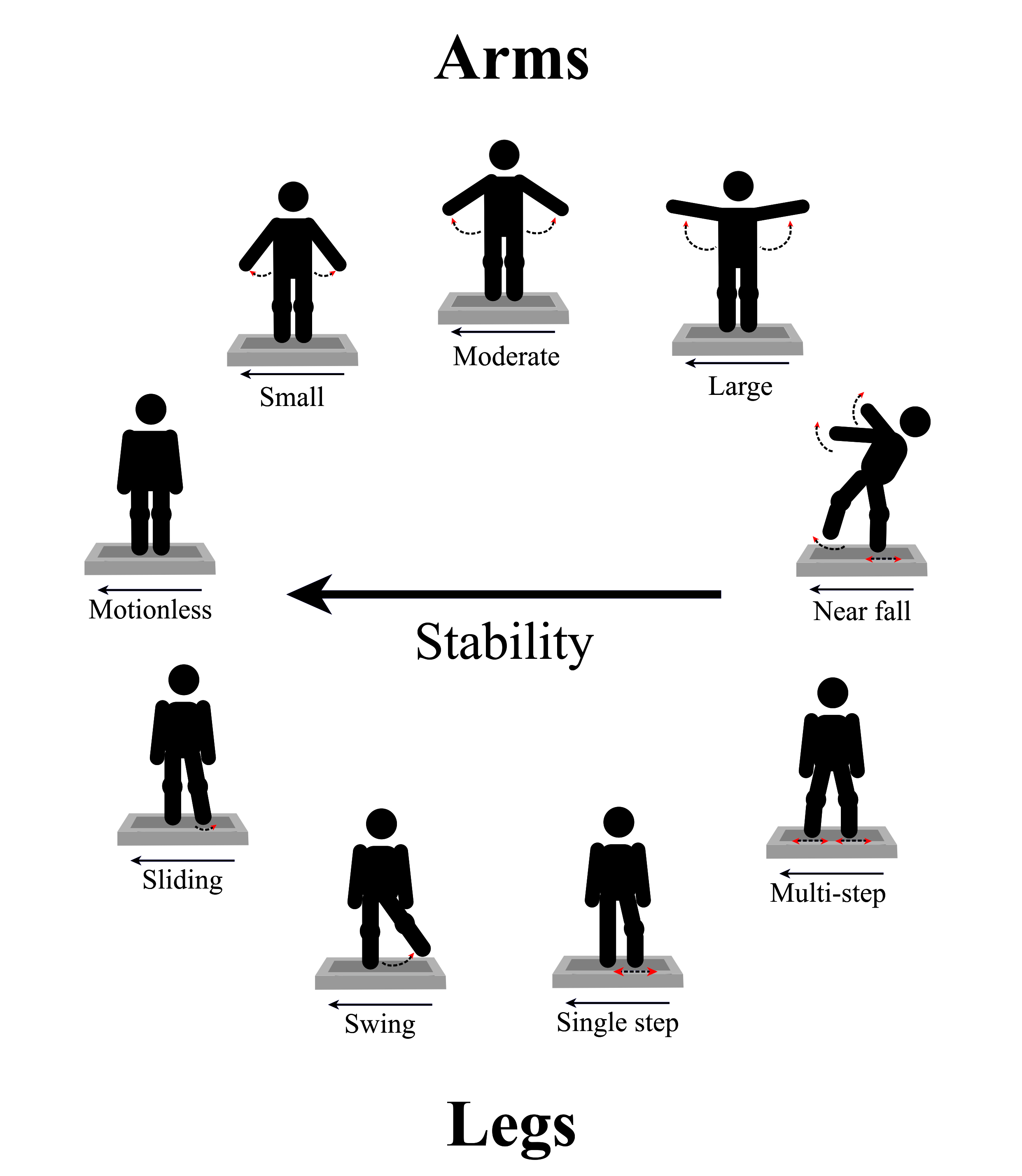 | 4 | **Small amplitude**. Minor shoulder abduction, with contact loss between the hand(s) and the leg(s) up to about 10 cm. |
| 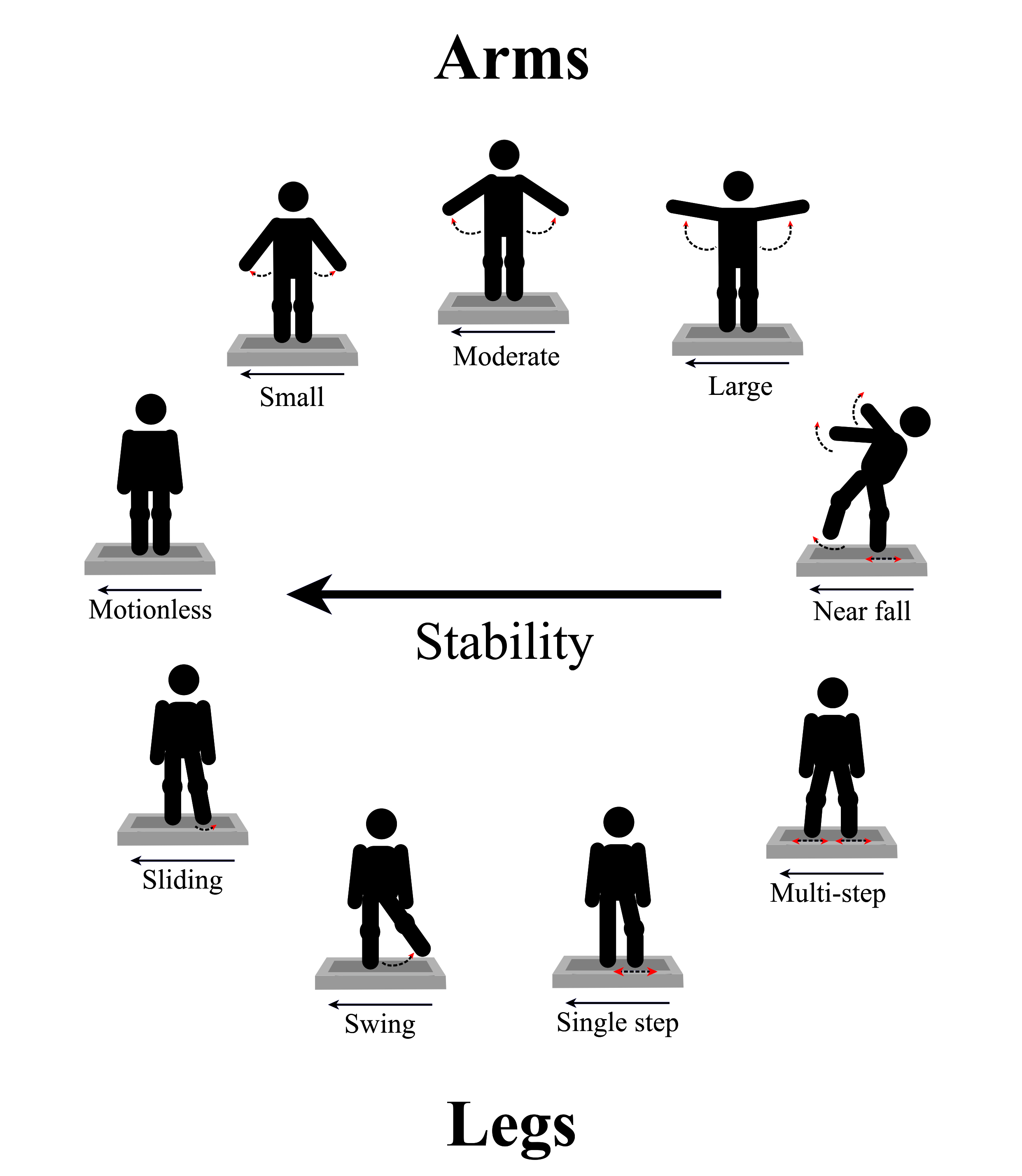 | 5 | **Motionless**. Keeping the hands in contact with the legs while recovering body equilibrium. |
| Leg movements | | |
| Representation | Score | Description |
| 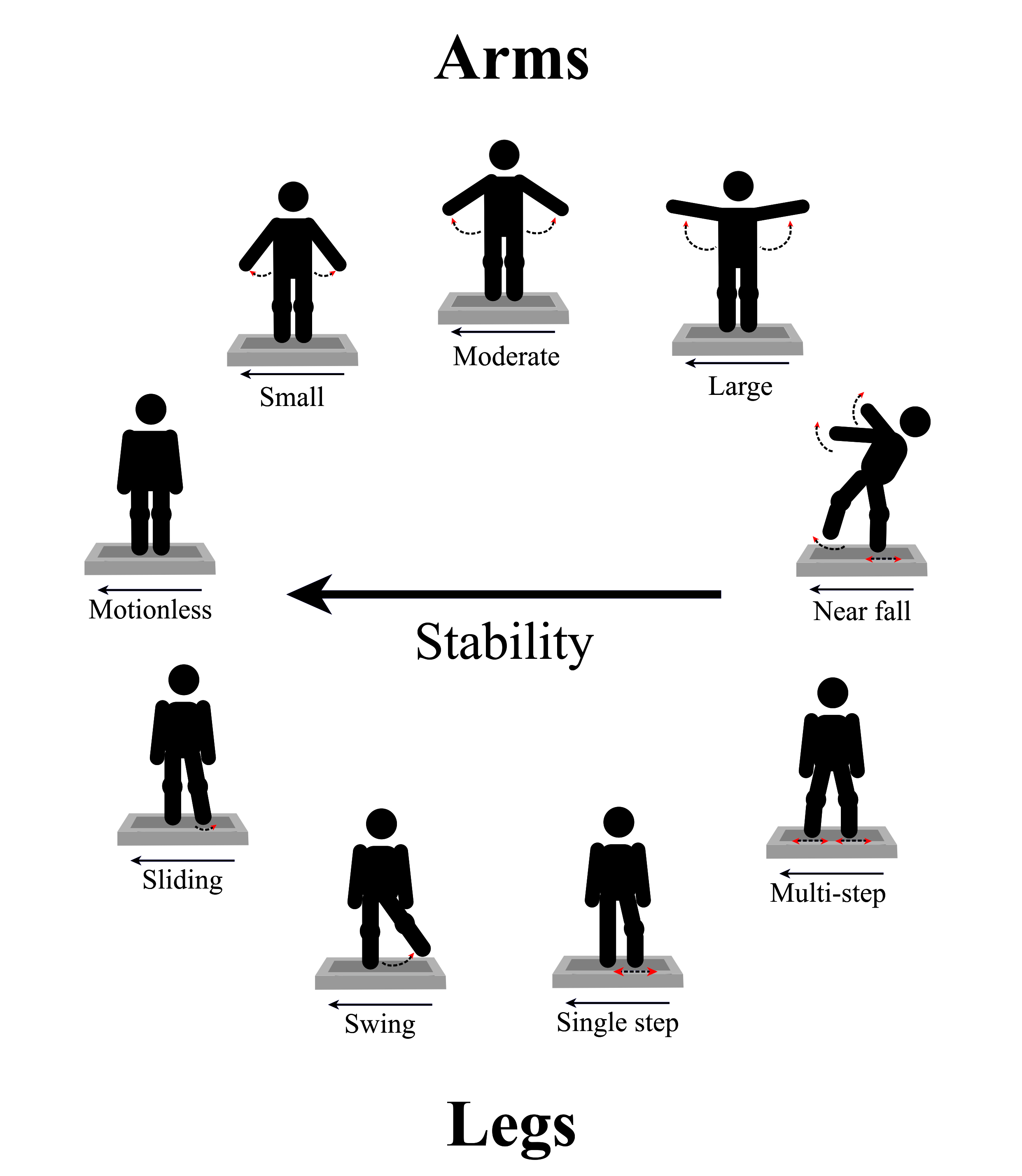 | 1 | **Near fall**. Being supported by the safety device or grasping a near surface, regardless of leg movements. |
| 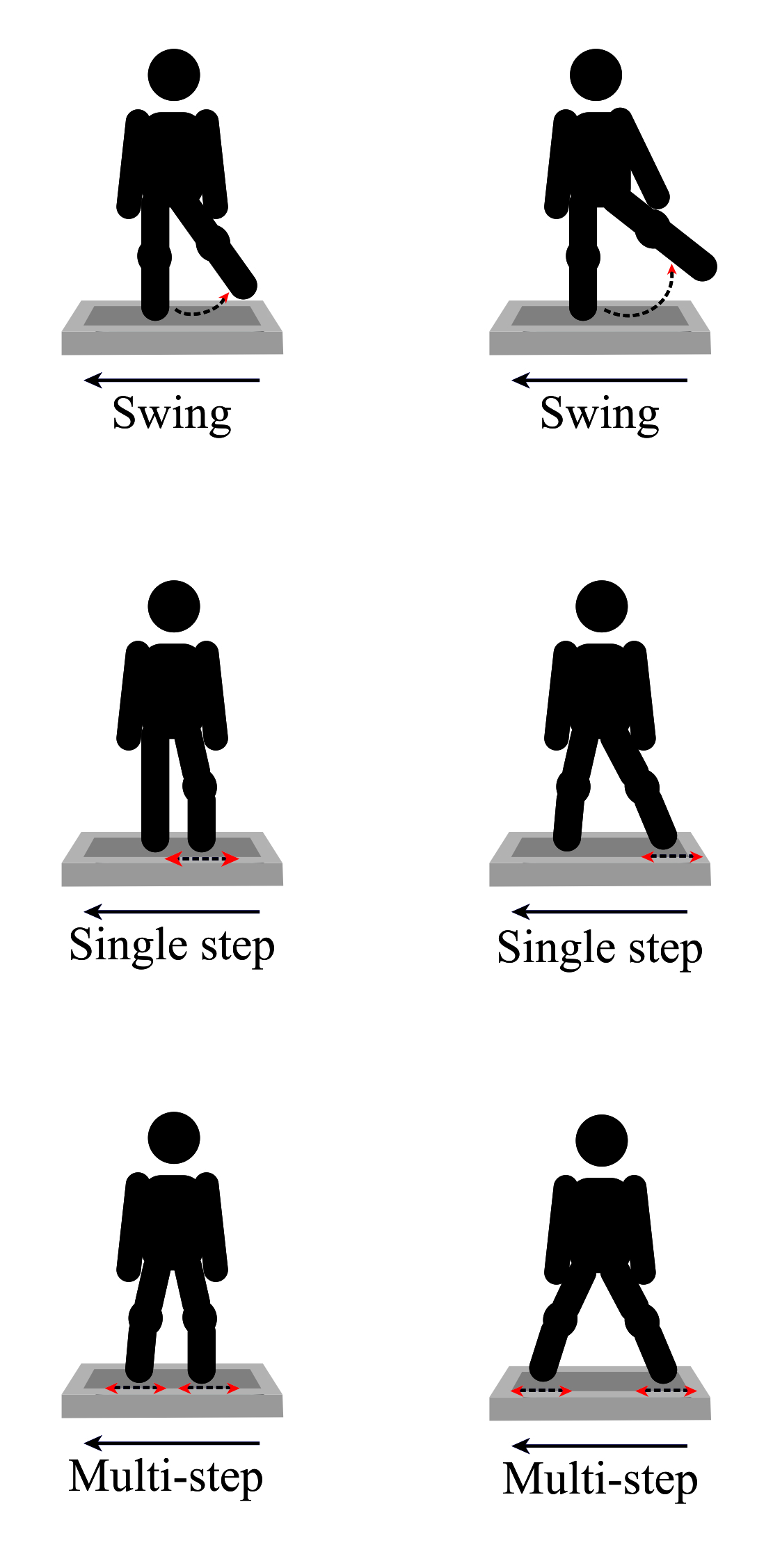 | 2 | **Multiple steps through large increment of the support base**. Changing the support base for balance recovery through two or more steps, regardless the stepping pattern, with total displacement larger than 15 cm. |
| 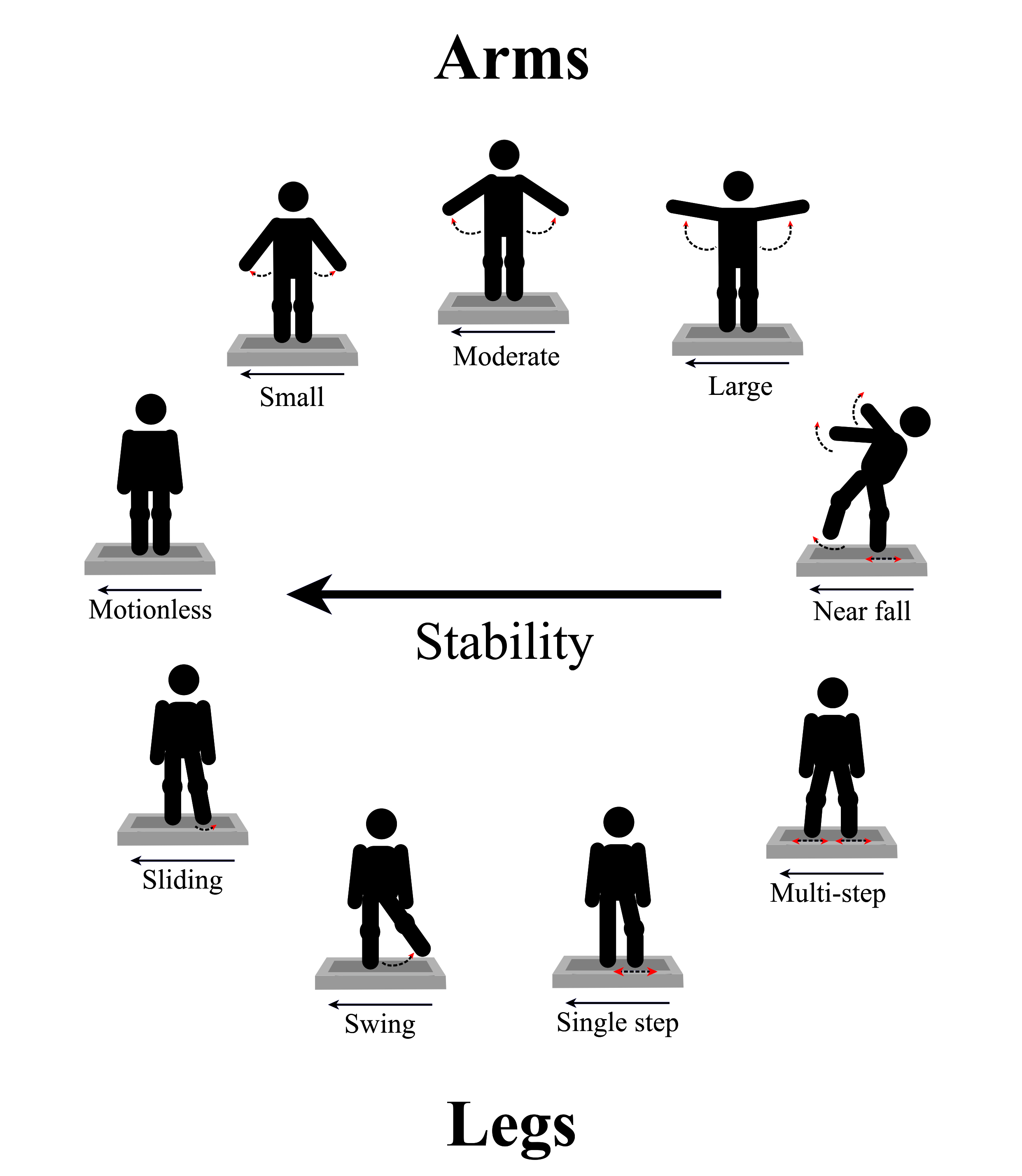 | 3 | **Multiple steps through small increment of the support base**. Changing the support base for balance recovery through two or more steps, regardless the stepping pattern, with total displacement equal to or smaller than 15 cm. |
| 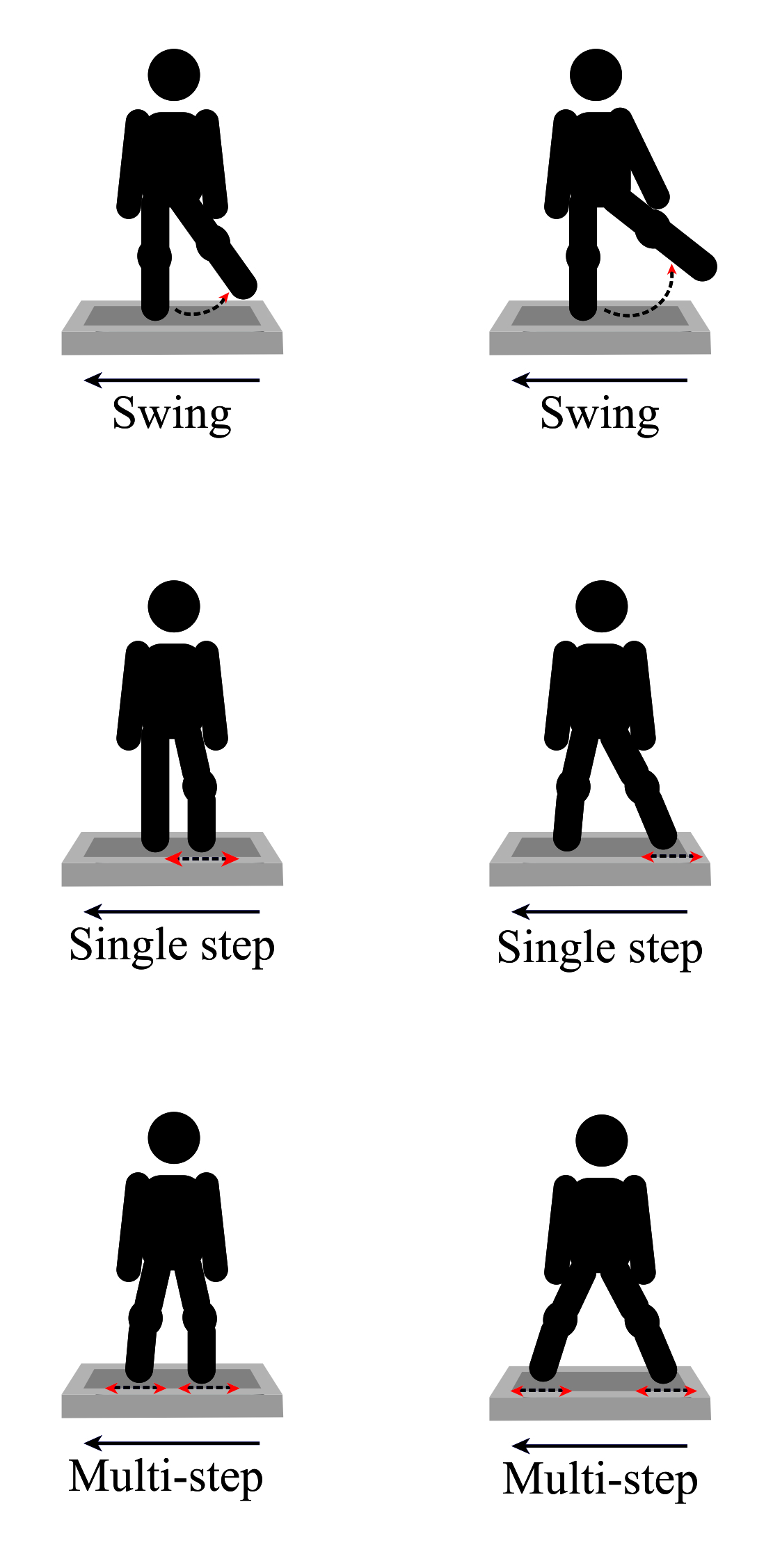 | 4 | **Single step through large increment of the support base**. Balance recovery through a single step, regardless the stepping pattern, with stepping amplitude larger than 15 cm. |
| 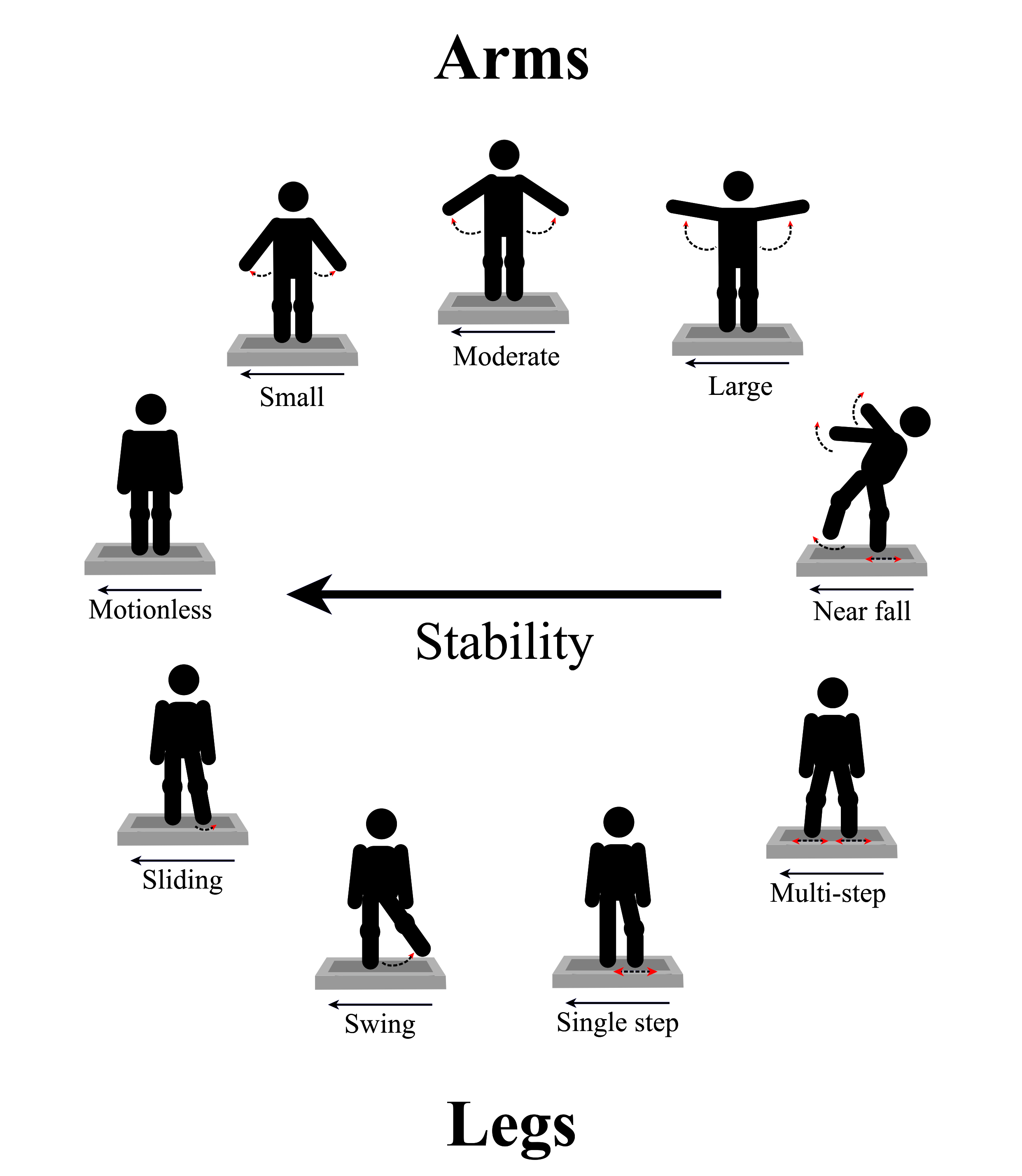 | 5 | **Single step through small increment of the support base**. Balance recovery through a single step, regardless the stepping pattern, with stepping amplitude equal to or smaller than 15 cm. |
| 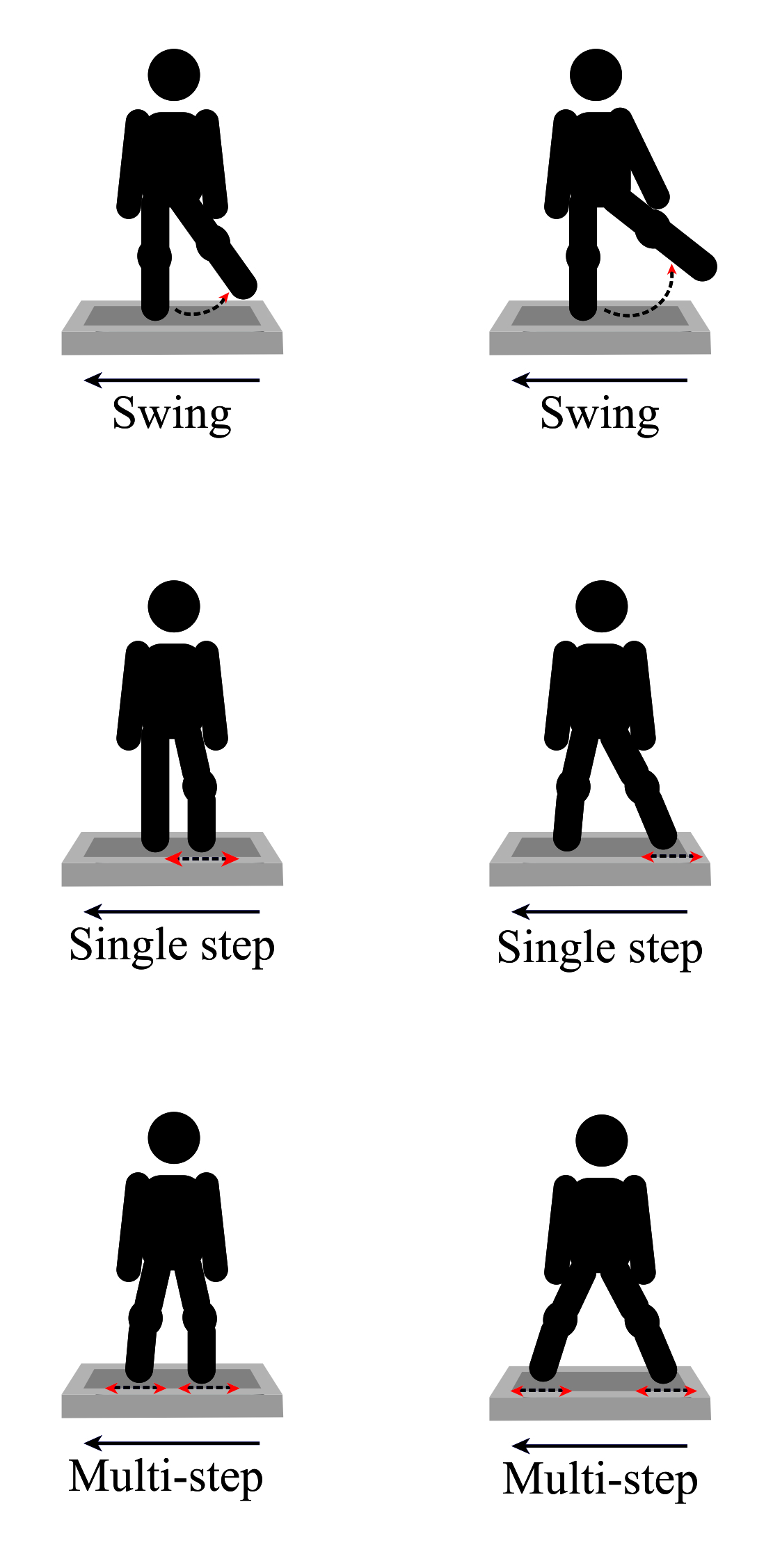 | 6 | **Leg swing with large amplitude**. Swinging one leg outward for counter-weighting lateral body leaning while supporting the whole body on the other leg, with movement amplitude larger than 15 cm. |
| 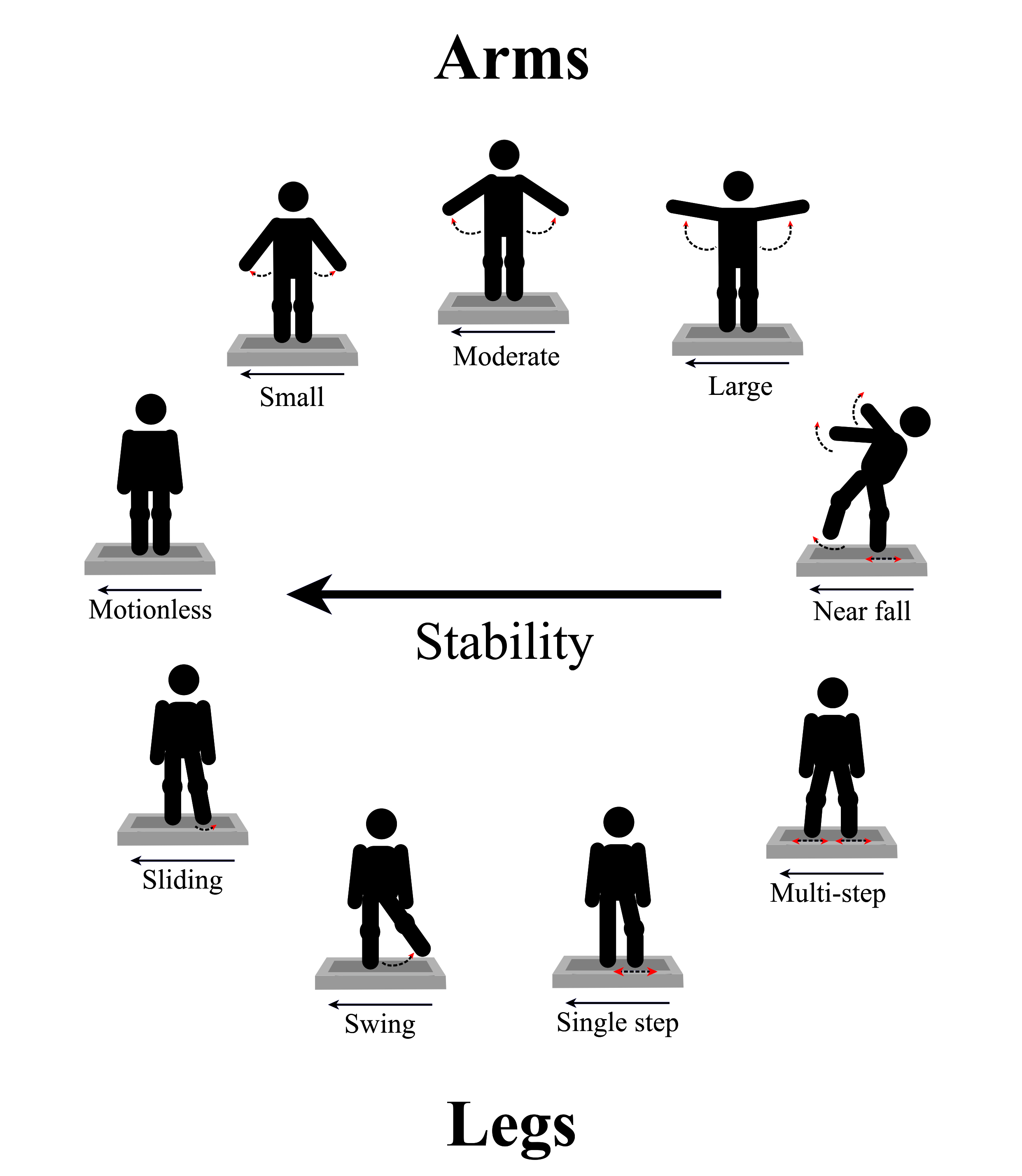 | 7 | **Leg swing with small amplitude**. Swinging one leg outward for counter-weighting trunk movements while supporting the whole body on the other leg, with movement amplitude equal to or smaller than 15 cm. |
| 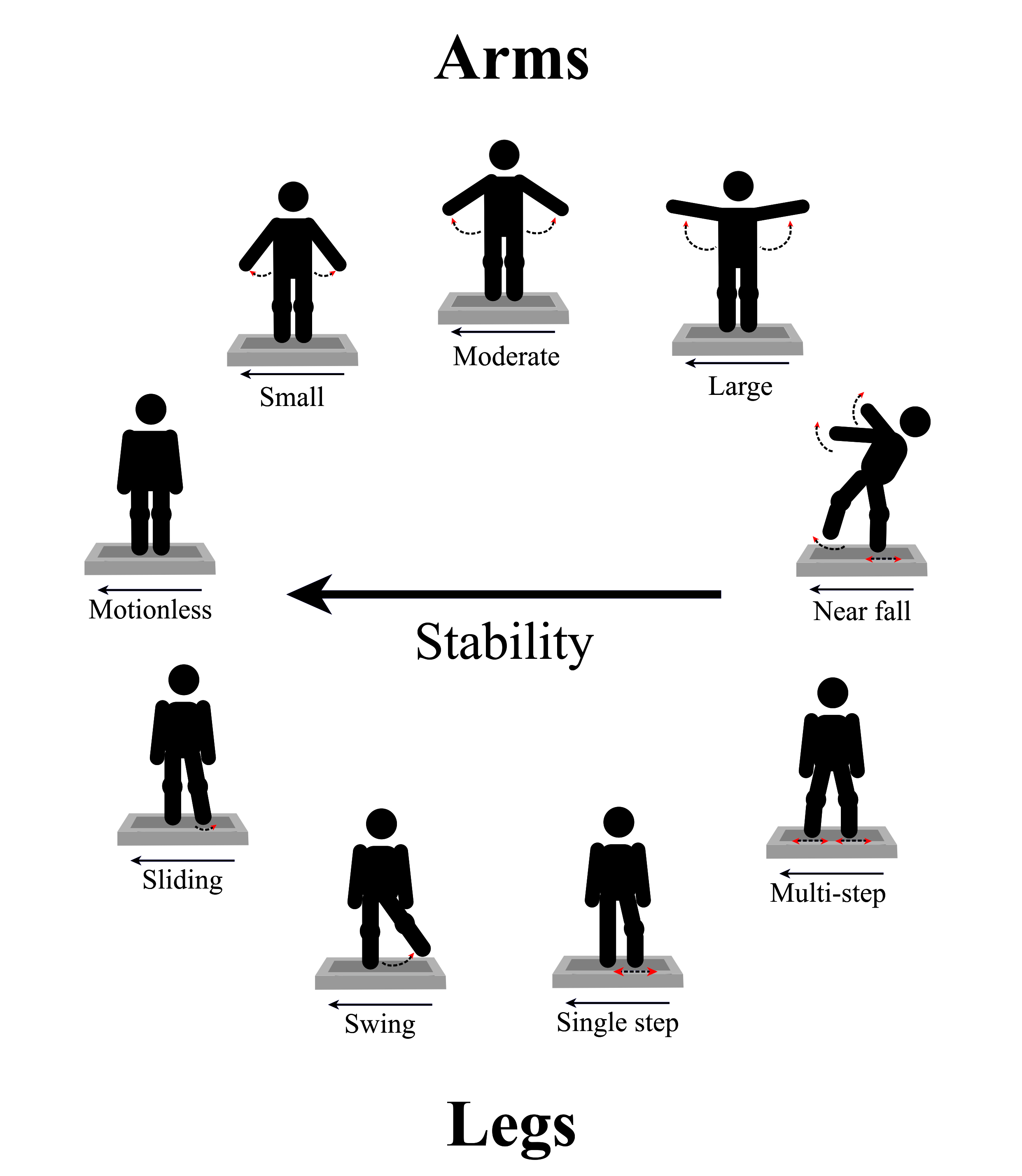 | 8 | **Sliding**. Short one-foot or two-feet outward sliding over the support base (no feet-ground contact loss), not crossing the 5-cm mark on the platform, or short (few centimeters) one-foot rising above the ground landing at about the place . |
| 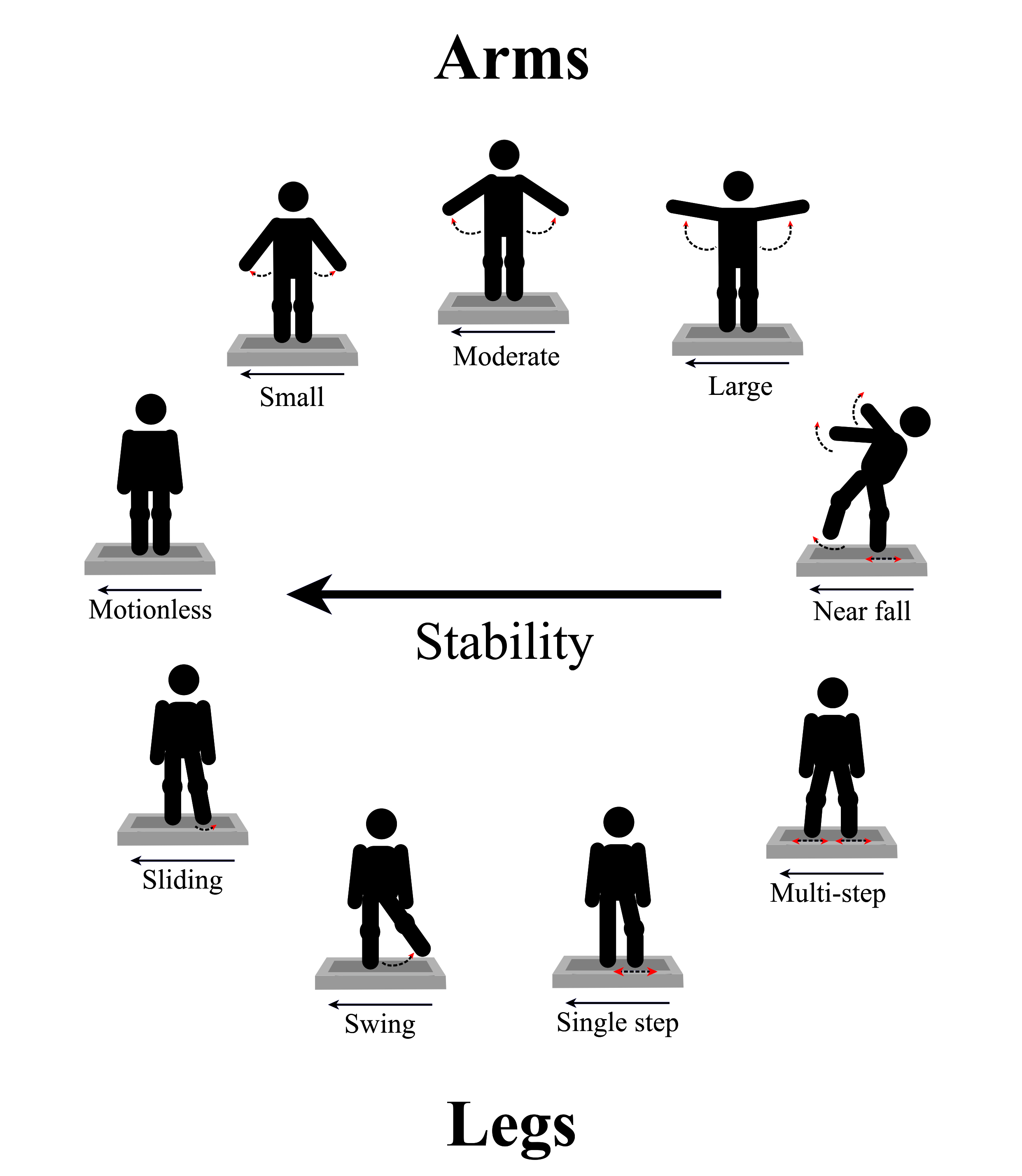 | 9 | **Motionless**. Balance recovery keeping the feet in place. |
